# Introduction to the Mymaridae (Hymenoptera) of Bangladesh

**DOI:** 10.3897/zookeys.675.12713

**Published:** 2017-05-22

**Authors:** John T. Huber, Nurul Islam

**Affiliations:** 1 Natural Resources Canada c/o AAFC, 960 Carling Ave., Ottawa, ON, K1A 0C6, Canada; 2 Forest Entomology Laboratory, Faculty of Forestry, University of Toronto, Toronto,ON, M5S 3B3, CANADA

**Keywords:** Mymaridae, Bangladesh, identification key, list of genera

## Abstract

An identification key to the 15 genera of Mymaridae found so far in Bangladesh is given, based on about 520 specimens collected using yellow pan traps placed in agricultural habitats and at the edge of ponds, mainly at Bangabandhu Sheikh Mujibur Rahman Agricultural University, Gazipur. Species already reported from Bangladesh are listed and three more are added: *Acmopolynema orientale* (Narayanan, Subba Rao & Kaur), *Himopolynema hishimonus* Taguchi, and *Mymar pulchellum* Curtis.

## Introduction

Ten named species of Mymaridae (Hymenoptera), representing four genera, have been recorded from Bangladesh:

*Anagrus flaveolus* Waterhouse (Kamal et al. 1993, Sahad and Hirashima 1984), almost certainly a misidentification of *A. nilaparvatae* Pang & Wang (Triapitsyn 2014);

*Anagrus incarnatus* Haliday (Sahad and Hirashima 1984, Gurr et al. 2011), this is likely a misidentification of *A. nilaparvatae* (Chiappini 2002: 236);

*A. nilaparvatae* (Triapitsyn and Berezovskiy 2004, Triapitsyn 2015);

*A. optabilis* (Perkins) (Kamal et al. 1993, Sahad and Hirashima 1984, Trjapitzin 1996);

*A. perforator* (Perkins) (Sahad and Hirashima 1984);

*Lymaenon uttardeccanus* [sic] (Mani & Saraswat) (Sahad and Hirashima 1984) but considered likely to be a misidentification of *Gonatocerus longicornis* Nees by Zeya and Hayat (1995);

*L. devitatakus* (Mani & Saraswat) (Sahad and Hirashima 1984) but considered likely to be a misidentification of L. *pahlgamensis* Narayanan by Zeya and Hayat (1995) and this, in turn, synonymized under *L. aureus* (Girault) by Triapitsyn (2013);

*L. narayani* Subba Rao & Kaur (Sahad and Hirashima 1984);

*L. munnarus* (Mani & Saraswat) (Sahad and Hirashima 1984);

*Palaeoneura bagicha* (Narayanan, Subba Rao & Kaur) (Bhuiya et al. 1997).

In Bangladesh, Kamal et al. (1993) reared *A. nilaparvatae* (as “*flaveolus*”) and *A. optabilis* from the important rice pest *Nilaparvata lugens* (Stål) (Hemiptera: Delphacidae). Catling and Islam (2013) reported *Anagrus* sp. and *Gonatocerus* sp. from rice fields. No definite statements about deposition of voucher depositories are given for most of the previously recorded specimens. Gapud (1992) mentioned that there were no decent reference collections of insect pests and their natural enemies in any institution in Bangladesh. His list of 11 species of Mymaridae were all from Indian records. However, voucher specimens of at least one of the species (*P. bagicha*) mentioned in the literature above may be in the Department of Zoology, University of Chittagong, Chittagong and the specimens (probably in Oudeman’s fluid) collected by Kamal et al. (1993) are likely in the Bangladesh Rice Research Institute, Gazipur.

We present an identification key to the genera and illustrate the head, antennae, and wings of females of 13 of them (females of *Dicopus* and *Cosmocomoidea* not yet collected).

## Methods

Yellow pan traps three-quarters filled with water and a few drops of liquid detergent to break the surface tension were placed in small plot experimental fields and at the edge of ponds during June and August, 2007 at Bangabandhu Sheikh Mujibur Rahman Agricultural University (BSMRAU), Gazipur and, for the pond traps, at Kalni Village, Gazipur. A few specimens were also collected in December, 2008, and January, 2009. The plots were planted with lady’s finger [okra]—*Abelmoschus esculentus* (L.) Moench (Malvaceae), brinjal [eggplant]—*Solanum melongena* L. (Solanaceae), white gourd—*Benincasa hispida* Cogn. (Cucurbitaceae), amaranthus—*Amaranthus tricolor* L. (Amaranthaceae), long bean—Vigna unguiculata (L.) Walp subsp. sesquipedalis (Fabaceae), and hyacinth bean—Lablab purpureus L. subsp. bengalensis (Fabaceae). Trap catches were washed and preserved in 70% ethanol. All Mymaridae were later extracted from the ethanol, critical-point dried, and mounted on cards. Representative specimens of all but two of the genera were slide mounted in Canada balsam, using the method described in Huber (2015). Photographs of the head, antenna, and wings were taken with a ProgRes C14^plus^ digital camera attached to a Nikon Eclipse E800 compound microscope, and a selection of the resulting layers combined electronically and edited in Zerene Stacker^TM^. Specimens are deposited in the Canadian National Collection of Insects, Arachnids and Nematodes, Ottawa, Ontario, Canada, and the University of Rajshahi, Motihar, Rajshahi, Bangladesh. Abbreviations used in the key are: fl_x_ for funicle segment, and mps for multiporous plate sensilla.

## Results

The breakdown of the ≈ 520 card- and slide-mounted specimens is approximately as follows (some specimens of the two most commonly collected genera, *Anagrus* and *Lymaenon*, were kept in gelatin capsules): *Acmopolynema* 2, Anagrus (Anagrus) + Anagrus (Paranagrus) 160, *Anaphes* 62, *Camptoptera* 6, *Cosmocomoidea* 1, *Dicopus* 2, *Erythmelus* 2, *Gonatocerus* 53, *Himopolynema* 6, *Lymaenon* 140, *Mymar* 27, *Palaeoneura* 3, Polynema (Polynema) + P. (Dorypolynema) 26, *Ptilomymar* 2, *Stethynium* 26. Two genera, *Cosmocomoidea* and *Dicopus*, are represented by one or two males only so are not included in the key.

### Key to genera. Females.

(Arrows on figures indicate many of the key features to be observed)

**Table:** 

1	Fore wing without membrane for over half its length, then widening suddenly into an oval membranous area with its apical half dark brown (Fig. 24); hind wing a short stalk without membrane (Figs 24, 25); face with toruli abutting transverse trabecula (Fig. 22); antenna with extremely long scape constricted medially and fl_2_ extremely long, about half the length of the funicle (Fig. 23)	***Mymar* Curtis**
–	Fore wing with membrane for all of its length, variously shaped and not infuscated with brown as above; face with toruli separated by at least one torular diameter from transverse trabecula; antenna not as above, the scape not as long and not constricted medially and fl_2_ not much different in length from remaining funicle segments	**2**
2(1)	Funicle 8-segmented (Figs 15, 19, 20), though fl_1_ may be very short and inconspicuous (Fig. 31)	**3**
–	Funicle with 7 or fewer (almost always 6) segments, if with 7 segments fl_2_ often minute, ringlike (Fig. 9)	**6**
3(2)	Face with a distinct subantennal groove extending from each torulus to mouth margin (Fig. 13); toruli separated by at least half a torular diameter from transverse trabecula (Figs 13, 19); hind wing relatively wide, the distance between anterior and posterior margins at most about the length of a setae on the wing membrane (Figs 16, 21)	**4**
–	Face without subantennal grooves; toruli abutting transverse trabecula (Fig. 30); hind wing extremely narrow, the distance between anterior and posterior margins at most about the length of a setae on the wing membrane (Fig. 32)	***Ptilomymar* Annecke & Doutt**
4(3)	Fore wing bare or almost so behind venation; subantennal grooves almost in contact with each other but if not then with distance between them at junction with mouth margin much less than half the distance from a groove to preorbital groove at lateral margin of face	***Cosmocomoidea* Howard**
–	Fore wing with at least one row of microtrichia, but usually with numerous scattered microtrichia behind venation; subantennal grooves with distance between them at junction with mouth margin at least half distance from a groove to preorbital groove at lateral margin of face	**5**
5(4)	Antenna with fl_2_ and fl_3_ longer than either fl_1_ or fl_4_ (Fig. 15); stigmal vein with apex oblique (Fig. 16); face with distance between antennal grooves less than distance between subantennal groove and preorbital groove (Fig. 13); ocellar triangle with 2 setae between posterior ocelli (Fig. 14)	***Gonatocerus* Nees**
–	Antenna with fl_2_ and fl_3_ subequal to either fl_1_ or fl_4_ (Figs 19, 20); stigmal vein with apex truncate (Fig. 21); face with distance between antennal grooves equal to or greater than distance between subantennal groove and preorbital groove (Fig. 19); ocellar triangle with 3 setae	***Lymaenon* Walker**
6(2)	Funicle 7-segmented (apparently 6-segmented in one genus because fl_2_ often ringlike [Fig. 9])	**7**
–	Funicle 6-segmented	**8**
7(6)	Head in anterior view quite wide ventrally, the genae only slightly converging; mandibles directed medially, their apices crossing each other, the head not appearing beaklike; antenna with fl_2_ ringlike (Fig. 9); fore wing evenly wide along its length distal to venation and distinctly curved near apex (Fig. 10); gaster separated from propodeum by a distinctly narrow petiole, the mesophragma thus not extending posteriorly into gaster	***Camptoptera* Förster**
–	Head in anterior view quite narrow ventrally, the genae strongly converging; mandibles directed ventrally and narrowing apically, their apices usually not crossing each other and giving head a beak-like appearance; antenna with fl_2_ about as long as preceding and following segments; fore wing much narrower medially along much of its length distal to venation then distinctly widening near apex; gaster widely joined to propodeum by a wide petiole barely distinguishable from propodeum or gaster, so mesophragma projecting posteriorly well into gaster	***Dicopus* Enock**
8(6)	Metasoma with petiole wide, inconspicuous, not longer than wide	**9**
–	Metasoma with petiole narrow, conspicuous, and clearly longer than wide	**12**
9(8)	Face with distinct subantennal groove extending from each torulus to mouth margin (Fig. 33); antenna with clava 3-segmented (Fig. 33); fore wing with distinct lobe posterior to and just distal to apex of stigmal vein (Fig. 34)	***Stethynium* Enock**
–	Face without subantennal grooves; antenna with clava 1-segmented (Figs 4, 11) or 2 segmented (Fig. 7); fore wing usually without or with only a slight lobe (Figs 5, 8), rarely with a more distinct lobe posterior to and just distal to apex of stigmal vein (Fig. 27)	**10**
10(9)	Mandibles fully developed and crossing each other when closed, with 3 teeth; head in lateral view with eye clearly separated from back of head by distinct gena	**11**
–	Mandibles greatly reduced to minute stubs without teeth, and maxilla elongate; head in lateral view with eye in contact with back of head, the gena almost entirely absent; fore wing membrane rather sparsely and unevenly covered with microtrichia concentrated mainly in apical half of wing beyond venation apex (Fig. 12)	***Erythmelus* Enock**
11(10)	Vertex with ocellar triangle surrounded by a stemmaticum (seen as white lines) (Fig. 4); clava in lateral view usually asymmetrical, with its dorsal margin strongly curved and ventral margin straight (Fig. 4); fore wing narrow, without marginal and medial spaces and without socketed seta at apex of retinaculum	***A.* (*Anagrus* Haliday)** and ***Anagrus* (*Paranagrus* Perkins)**
–	Vertex with ocellar triangle not surrounded by a stemmaticum; clava in lateral view symmetrical, with both dorsal and ventral margins equally curved (Figs 6, 7); fore wing with marginal and medial spaces and with a socketed seta at apex of retinaculum (Fig. 8)	***Anaphes* Haliday**
12(8)	Propodeum medially either with a single carina, at least near posterior margin, or apparently without carinae	**13**
–	Propodeum medially with two submedian carinae, either forming a V or closely parallel posteriorly then diverging near dorsellum to form a Y, or bulging medially to form an oval	**14**
13(12)	Fore wing with posterior margin behind venation not or scarcely lobed (Fig. 29)	***P.* (*Polynema* Haliday)** and ***Polynema* (*Dorypolynema* Subba Rao)**
–	Fore wing with posterior margin behind venation distinctly lobed (Fig. 27)	***Palaeoneura* Waterhouse**
14(12)	Face with a small pit medial to each torulus (Fig. 17); antenna usually with funicle segments short (Fig. 17); fore wing without thickened setae	***Himopolynema* Taguchi**
–	Face without pits between toruli (Fig. 1); antenna usually with funicle segments, especially fl_2_, longer (Fig. 2); fore wing with at least a few, thickened blunt microtrichia mainly on the dark areas (Fig. 3)	***Acmopolynema* Ogloblin**

**Figures 1–3.**
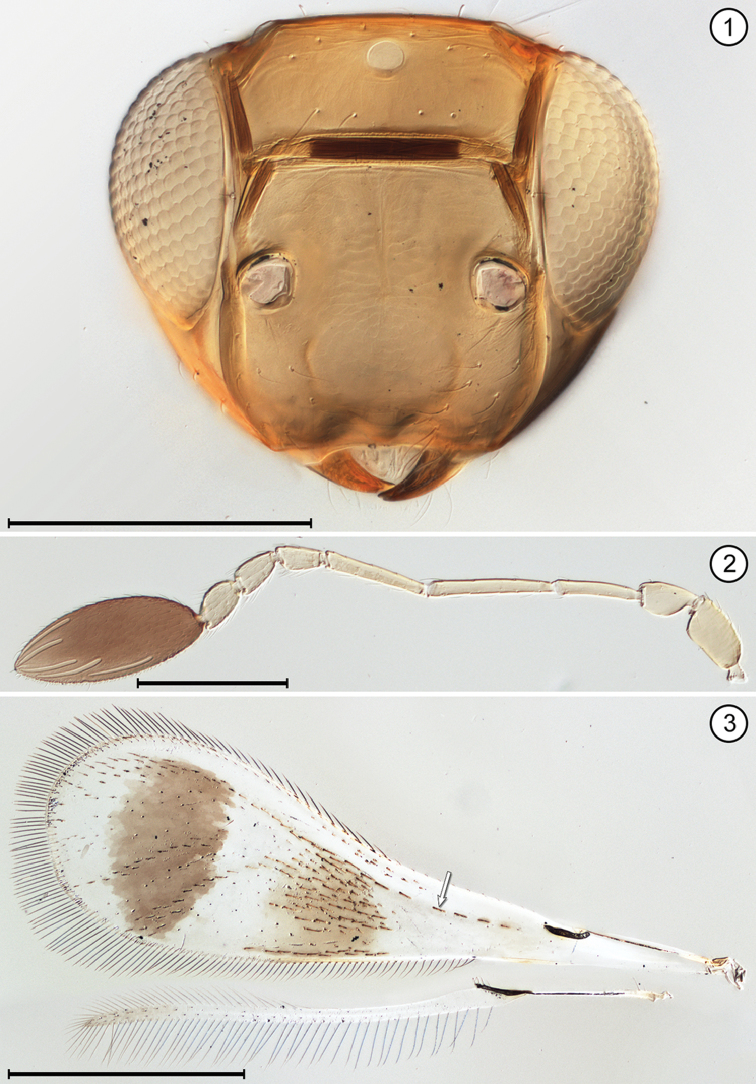
*Acmopolynema orientale*, female **1** head, anterior **2** antenna **3** wings. Scale bars for **1, 2** = 200 μm, **3** = 500 μm.

**Figures 4, 5.**
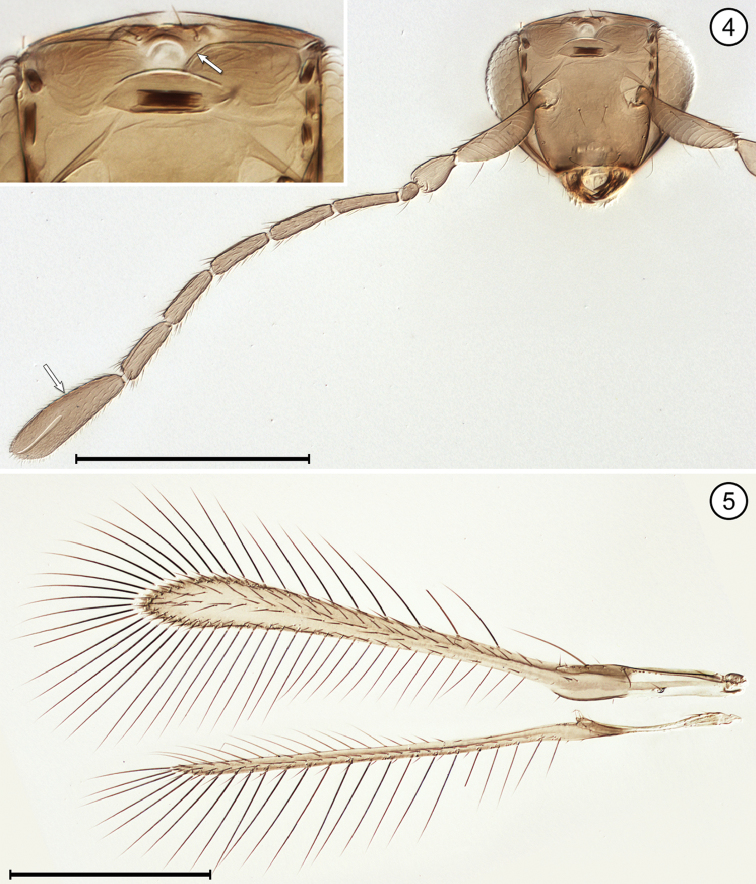
Anagrus (Anagrus) sp., female **4** head, anterior + antenna (inset is upper face and vertex) **5** wings. Scale bars = 200 μm.

**Figures 6–8.**
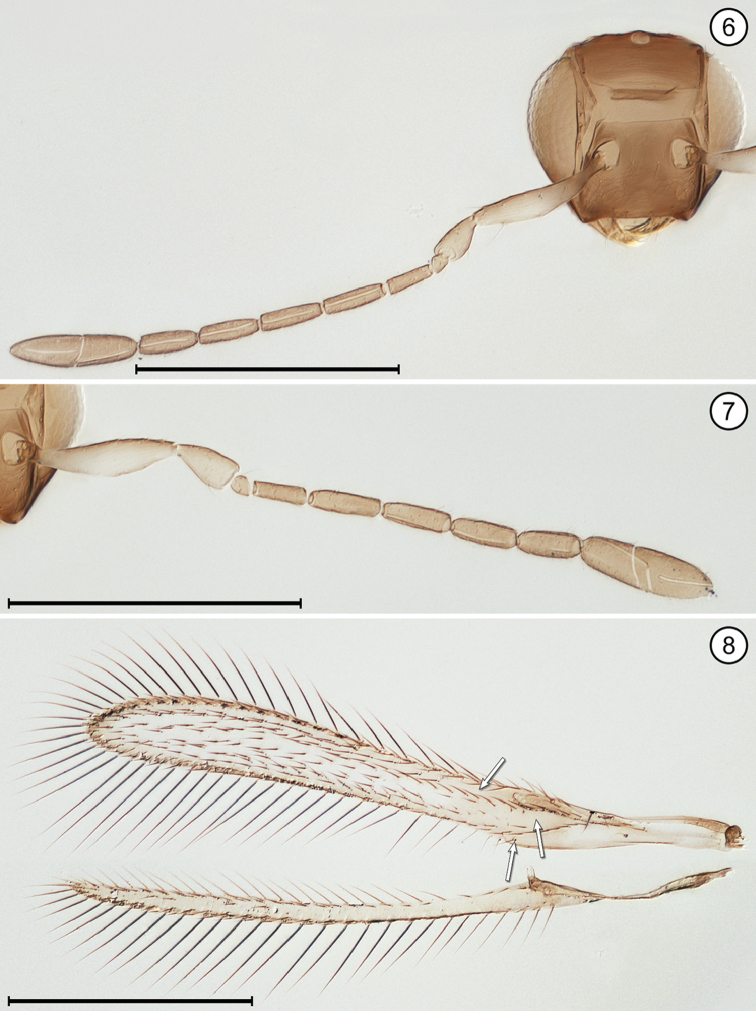
*Anaphes* sp., female **6** head, anterior + antenna **7** antenna **8** wings. Scale bars = 200 μm.

**Figures 9, 10.**
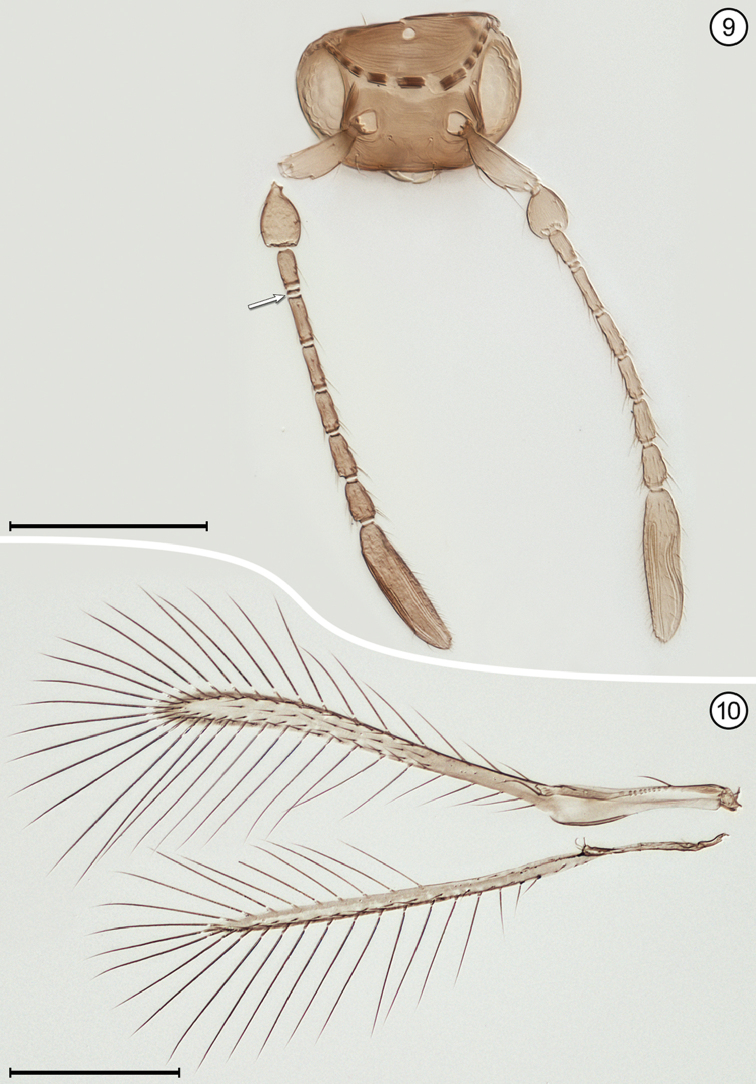
*Camptoptera* sp., female **9** head, anterior + antennae **10** wings. Scale bars = 100 μm.

**Figures 11, 12.**
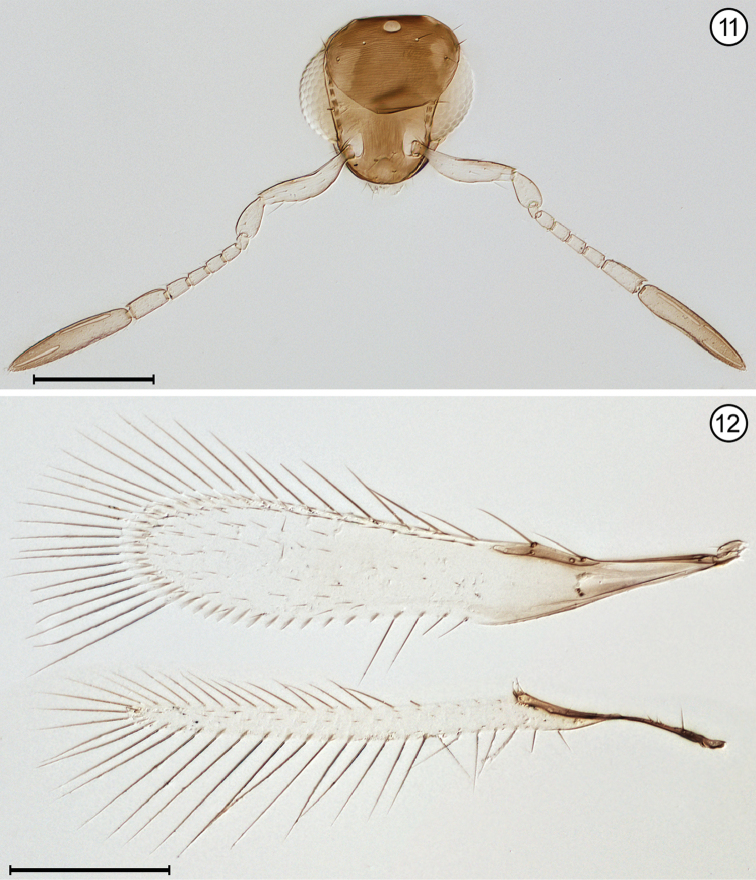
*Erythmelus* sp., female **11** head, anterior + antennae **12** wings. Scale bars = 100 μm.

**Figures 13–16.**
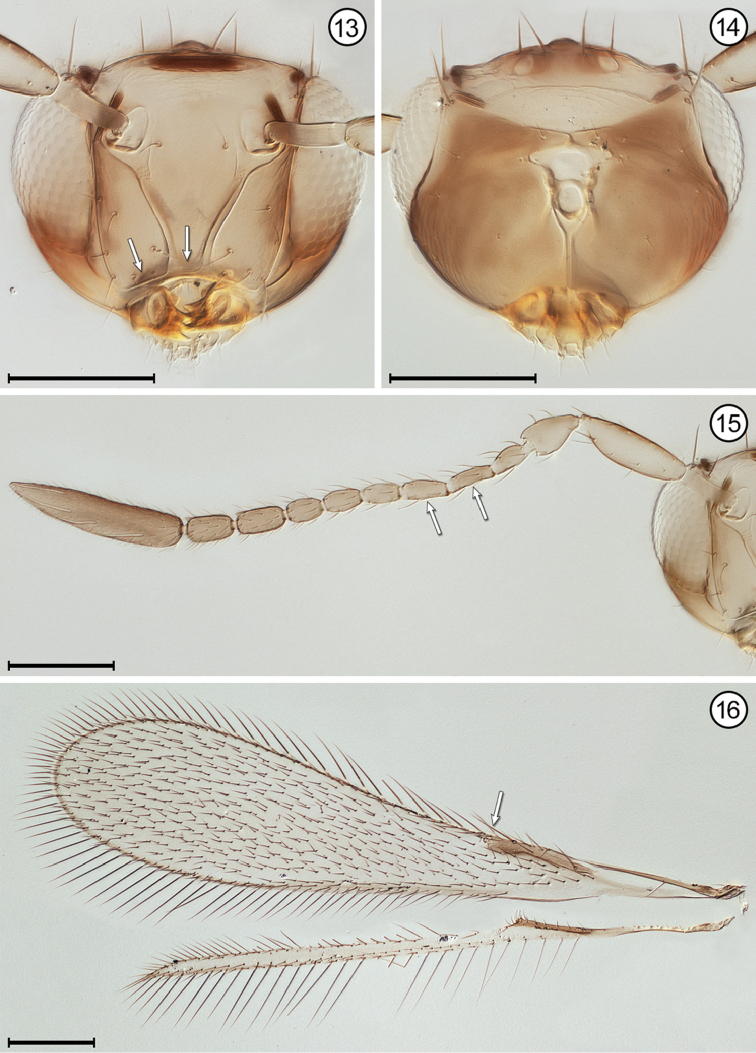
*Gonatocerus* sp., female **13** head, anterior **14** head, posterior **15** antenna **16** wings. Scale bars = 100 μm.

**Figures 17, 18.**
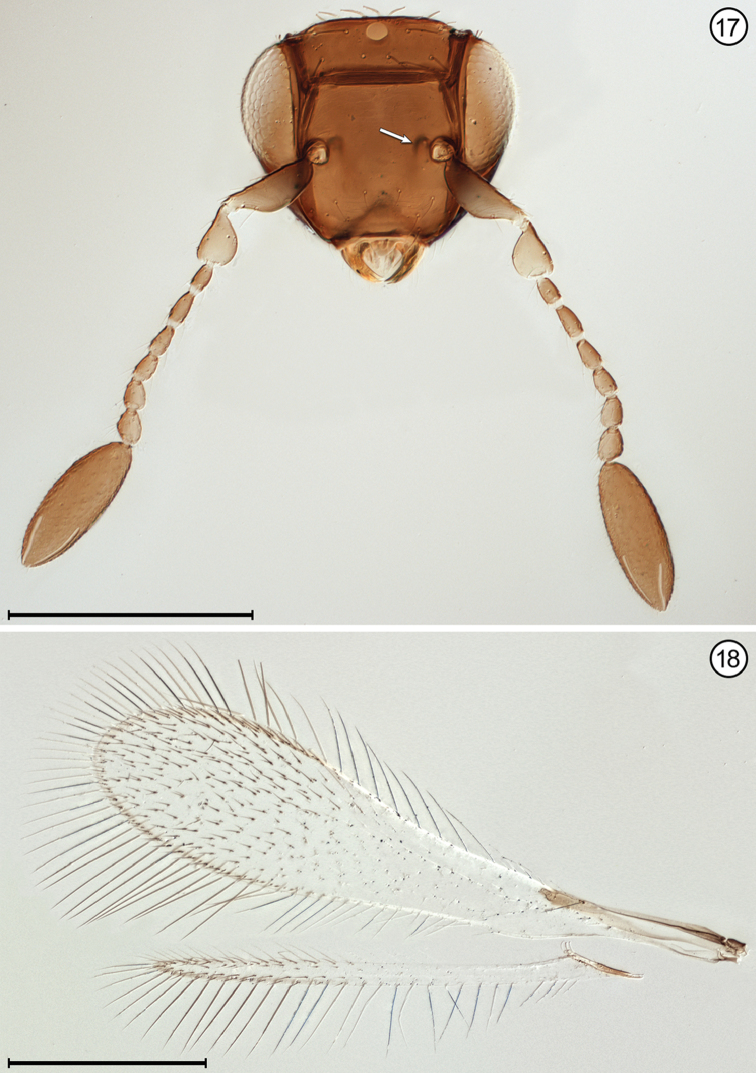
*Himopolynema hishimonus*, female **17** head, anterior + antennae **18** wings. Scale bars = 100 μm.

**Figures 19–21.**
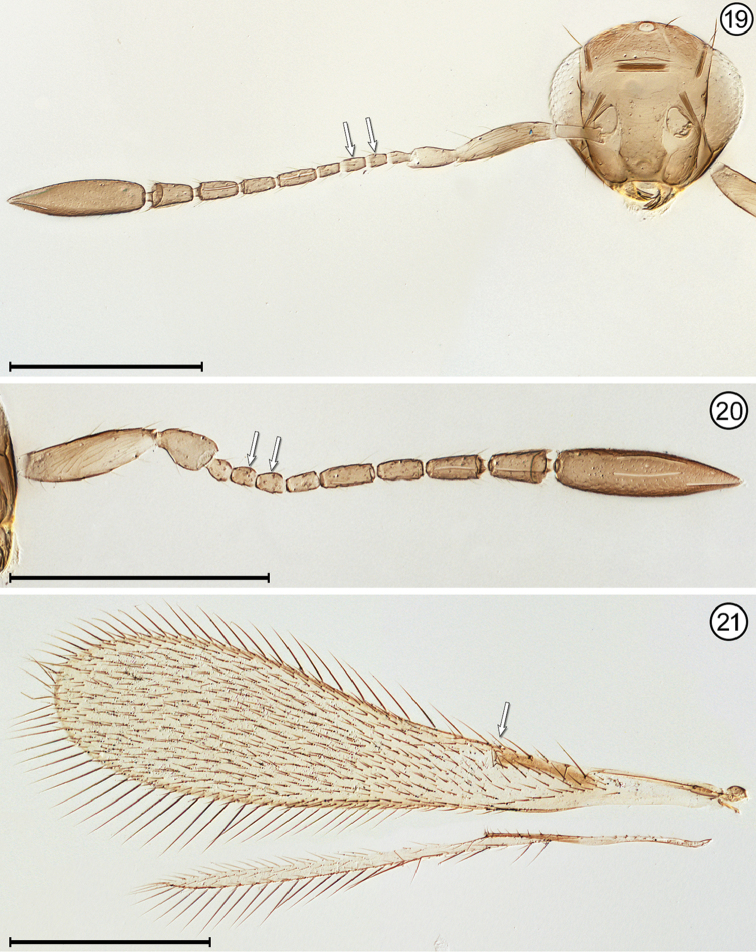
*Lymaenon* sp., female **19** head, anterior + antenna **20** antenna **21** wings. Scale bars = 200 μm.

**Figures 22–25.**
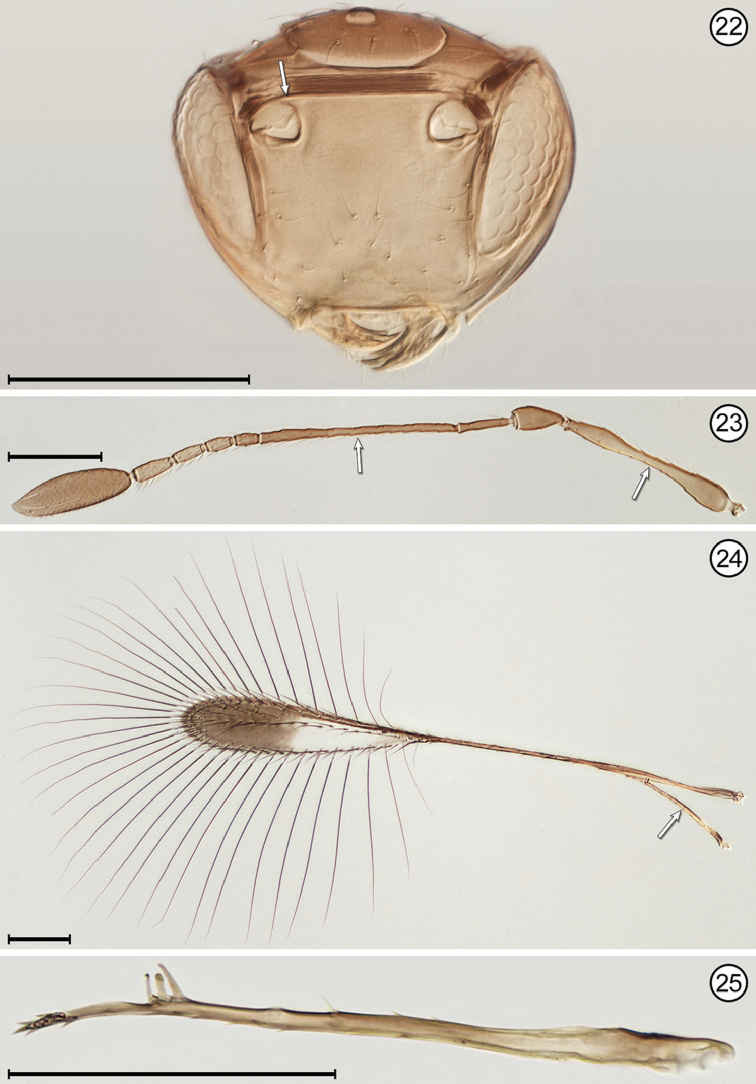
*Mymar pulchellum*, female **22** head, anterior **23** antenna **24** wings **25** hind wing. Scale bars = 100 μm.

**Figures 26, 27.**
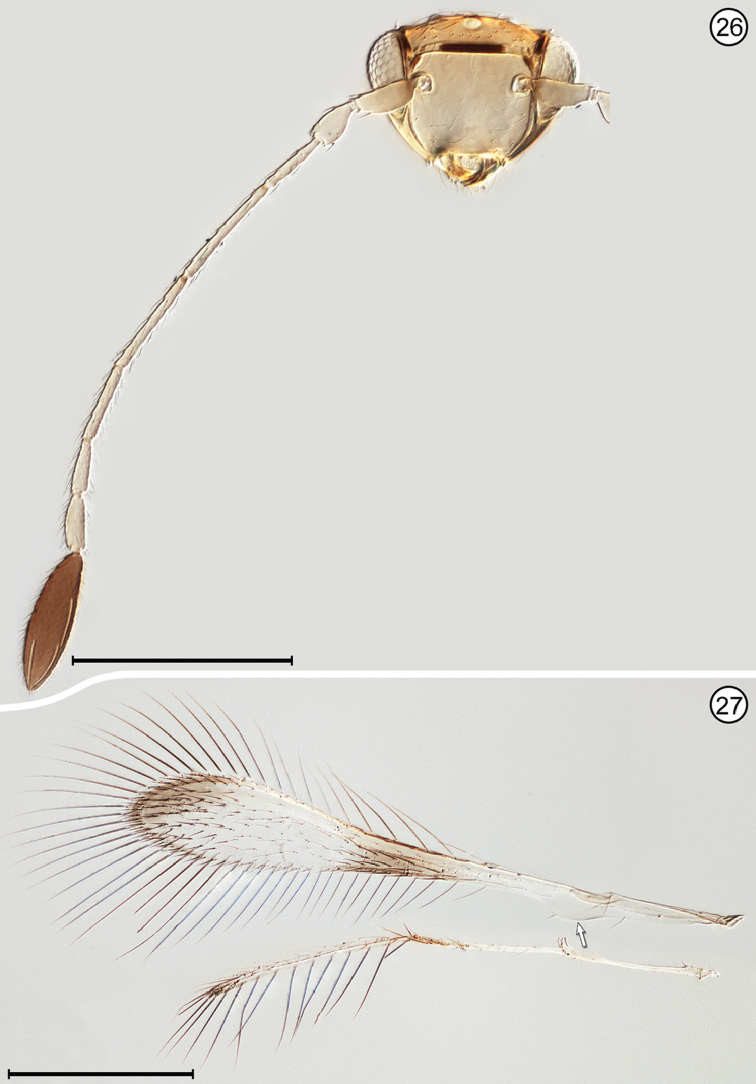
*Palaeoneura bagicha*, female **26** head, anterior + antenna **27** wings. Scale bars = 200 μm.

**Figures 28, 29.**
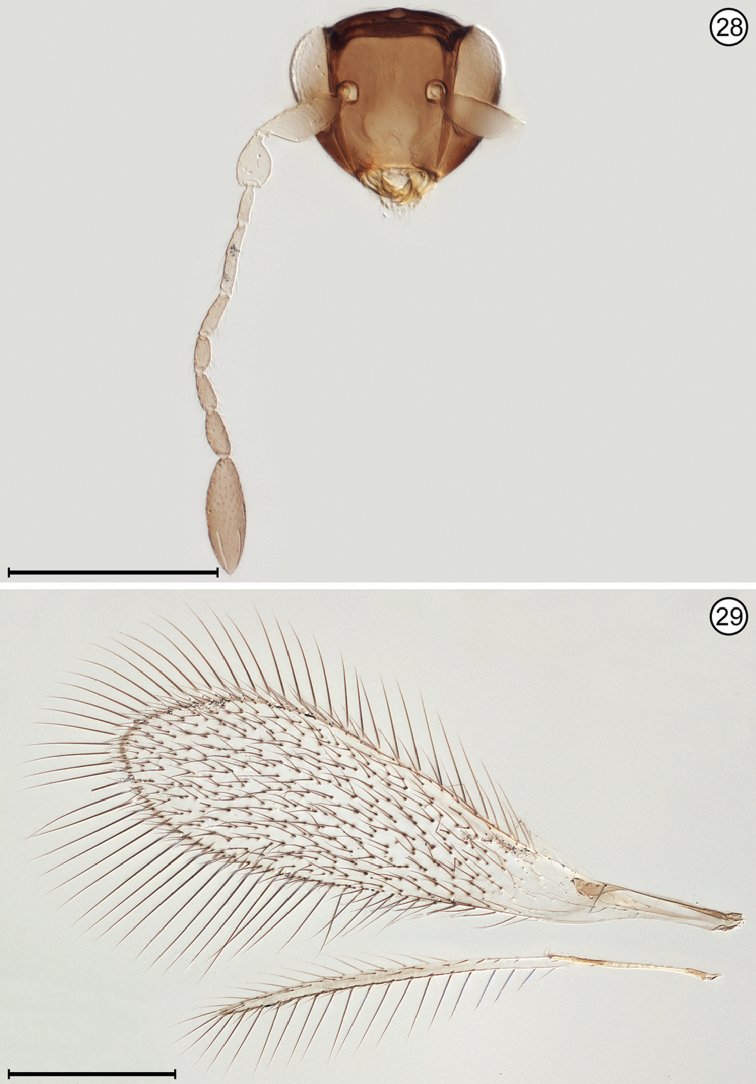
*Polynema* sp., female **28** head, anterior + antenna **29** wings. Scale bars = 200 μm.

**Figures 30–32.**
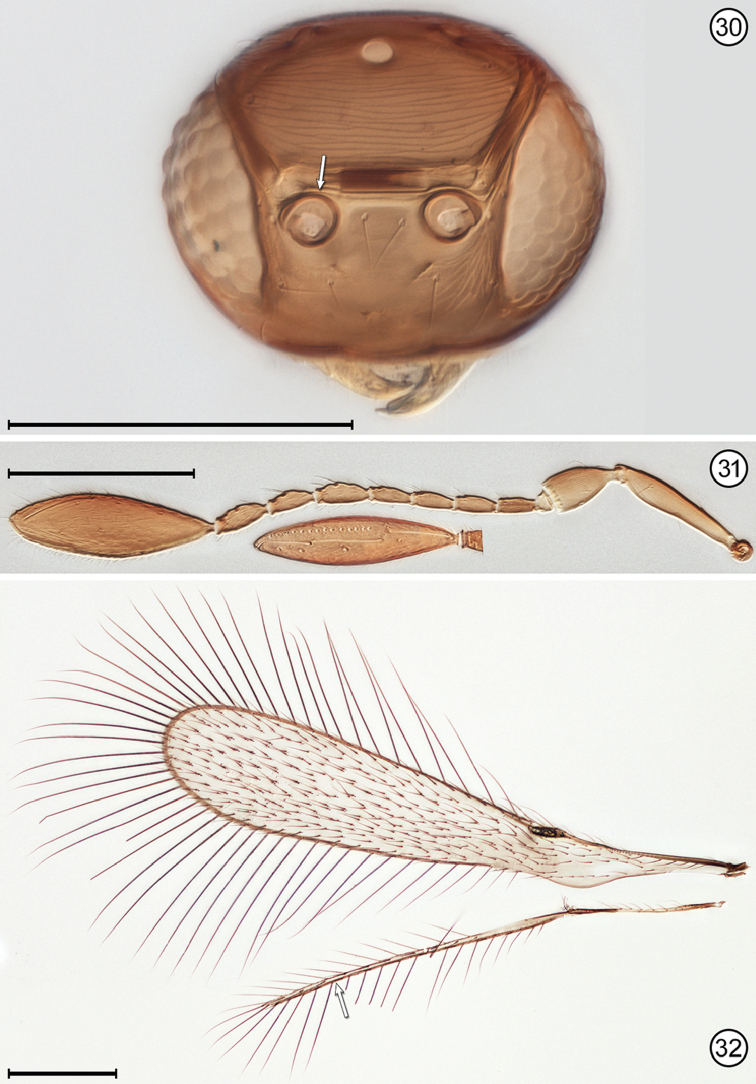
*Ptilomymar* sp., female **30** head, anterior **31** antenna (inset is clava, ventral) **32** wings. Scale bars = 100 μm.

**Figures 33, 34.**
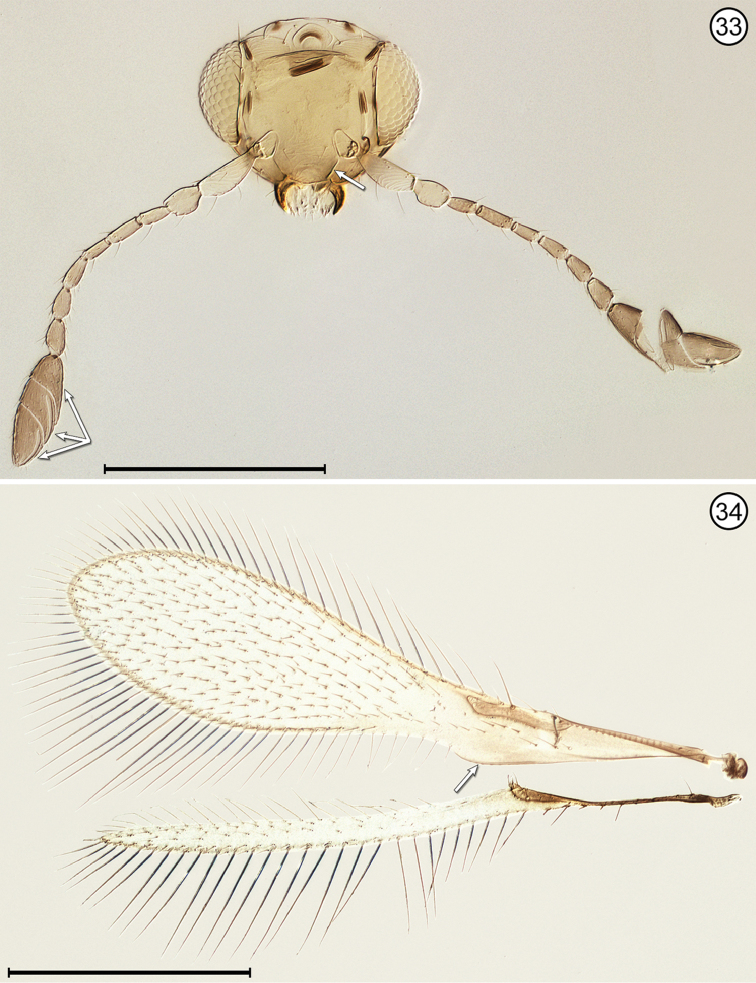
*Stethynium* sp., female **33** head, anterior + antennae **34** wings. Scale bars = 200 μm.

### Biology

Published host records exist for at least one species in most of the genera keyed above (Huber 1986). Cicadellidae and related families of Auchenorrhyncha (Hemiptera) are hosts for ten genera: *Acmopolynema*, Anagrus (Anagrus) + Anagrus (Paranagrus), *Cosmocomoidea*, *Gonatocerus*, *Himopolynema*, *Lymaenon*, *Mymar*, *Palaeoneura*, Polynema (Polynema) and *Stethynium* (Triapitsyn 2002). The remainder parasitize a variety of hosts. The principal host families only are listed here: *Anaphes* on Chrysomelidae and Curculionidae, *Erythmelus* on Tingidae and Miridae. *Camptoptera* and *Dicopus* have few or no published host records; they appear to parasitize Coleoptera and Psocoptera, respectively. Hosts are unknown for *Ptilomymar* which is closely associated with water and almost certainly parasitizes eggs of aquatic insects of some kind.

The various genera were collected in the following crops or habitats (not all specimens had associated plant names):

Amaranthus—*Amaranthus tricolor*. 5 genera: *Anagrus*, *Anaphes*, *Himopolynema*, *Gonatocerus*, *Mymar*.

Brinjal—*Solanum melongena*. 5 genera: *Anagrus*, *Anaphes*, *Lymaenon*, *Mymar*, *Stethynium*.

Lady’s finger—*Abelmoschus esculentus*. 6 genera: *Anagrus*, *Anaphes*, *Mymar*, *Palaeoneura*, *Polynema*, *Stethynium*.

Hyacinth bean/broad bean—*Lablab niger*. 6 genera: *Anagrus*, *Anaphes*, *Himopolynema*, *Lymaenon*, *Mymar*, *Polynema*.

Long bean—*Vigna unguiculata*. 4 genera: *Anagrus*, *Cosmocomoidea*, *Lymaenon*, *Palaeoneura*,

White gourd—*Benincasa hispida*. 6 genera: *Anagrus*, *Anaphes*, *Lymaenon*, *Mymar*, *Polynema*, *Stethynium*.

Pond (or near). 10 genera: *Anagrus*, *Anaphes*, *Camptoptera*, *Erythmelus*, *Gonatocerus*, *Himopolynema*, *Lymaenon*, *Mymar*, *Palaeoneura*, *Polynema*.

Ditch. 6 genera: *Acmopolynema*, *Anagrus*, *Camptoptera*, *Dicopus*, *Ptilomymar*, *Polynema*.

### Species and new specimen records

*Acmopolynema orientale* (Narayanan, Subba Rao & Kaur). BANGLADESH. **Rajshahi**: Serajganj, Krishnodia, 1.iii.2011, N. Islam, pan trap (1 female, CNC).

*Anagrus optabilis*. BANGLADESH. **Dhaka**: Joydebpur, 14.xii.2011, N. Islam, pan trap (2 females, CNC); BSMR Agricultural University, 19.vi2006–ii.2007, N. Islam, near pond (1 female, CNC).

*Himopolynema hishimonus* Taguchi. BANGLADESH. **Dhaka**: Joydebpur, 14.xii.2011, N. Islam, pan trap (1 female, CNC); Salna, BSMR Agricultural University, 18.vi-1.vii and 2–15.vii.2007, pan trap in *Amaranthus* field and hyacinth/broad bean field, N. Islam (5 females, CNC).

*Mymar pulchellum* Curtis. BANGLADESH. **Dhaka**: Salna, BSMR Agricultural University, 5–25.xii.2011, N. Islam, near pond (1 female, CNC).

*Palaeoneura bagicha* (Narayanan, Subba Rao & Kaur). BANGLADESH. **Dhaka**: Kalni, 2–5.vii.2007, N. Islam, pan trap in lady’s finger field (1 female, CNC).

## Discussion

Features useful for generic identification of most Mymaridae occur on the head, female antenna, and wings of specimens. Careful study of these structures requires well-mounted specimens on slides and/or good photographs. When these are available most specimens from a given country may be identified correctly to genus on this basis alone, often without having to examine other body parts. The generic identification key was carefully and deliberately constructed to demonstrate this. Features of the antenna and wings are also relatively easy to study on card- or point-mounted specimens. Only a few features of the mesosoma and metasoma were added, where necessary. However, additional features are certainly also useful and are needed when the fauna of an entire region is treated. Those additional features are, of course, widely used in all generic keys to Mymaridae, e.g., Ramesh Kumar et al. (2013) and are essential to define a genus properly.

At the generic level the almost unknown fauna of Mymaridae of Bangladesh, with 15 genera recorded here, is about 40% of the much better studied fauna of India. Ramesh Kumar et al. (2013) recorded about 140 species classified into 31 genera. Since then, Huber (2015) reclassified the species groups of *Gonatocerus* into separate genera and other genera (new to India, not yet recorded from Bangladesh) have been recorded (e.g., Triapitsyn 2014). bringing the number of Indian genera to almost 40. Many of the genera found in India almost certainly occur also in Bangladesh, as further collecting will undoubtedly reveal. The number of species in Bangladesh will be fewer than in India simply because it does not have the variety of ecosystems and elevational range of its far larger neighbour. At the species level, much more work would be needed to sort out and identify correctly the specimens collected in our study. This can only be done meaningfully in the context of more regional studies that include not only India but preferable the entire Oriental region and Palaearctic areas of eastern Asia.

The greatest number of genera collected was at pond edges. This is perhaps not surprising because it is a much more natural habitat, presumably with many more plant species and potential insect hosts (both terrestrial and aquatic) than experimental field plots planted with a single crop.
